# *NDN* and *CD1A* are novel prognostic methylation markers in patients with head and neck squamous carcinomas

**DOI:** 10.1186/s12885-015-1806-8

**Published:** 2015-10-30

**Authors:** Shama Virani, Emily Bellile, Carol R. Bradford, Thomas E. Carey, Douglas B. Chepeha, Justin A. Colacino, Joseph I. Helman, Jonathan B. McHugh, Lisa A. Peterson, Maureen A. Sartor, Jeremy MG Taylor, Heather M. Walline, Greg T. Wolf, Laura S. Rozek

**Affiliations:** 1 Department of Environmental Health Sciences, University of Michigan School of Public Health, Ann Arbor, MI USA; 2 Department of Otolaryngology, University of Michigan Medical School, Ann Arbor, MI USA; 3 Department of Computational Medicine and Bioinformatics, University of Michigan, Ann Arbor, MI USA; 4 Department of Pathology, University of Michigan Medical School, Ann Arbor, MI USA; 5 Department of Biostatistics, University of Michigan, School of Public Health, Ann Arbor, MI USA; 6 Department of Oral-Maxillofacial Surgery, University of Michigan Dental School, Ann Arbor, MI USA; 7 Department of Surgery, University of Michigan Medical School, Ann Arbor, MI USA; 8 1415 Washington Heights, Environmental Health Sciences 6630 SPH, Ann Arbor, MI 48109-2029 USA

**Keywords:** Head and neck cancer, Epigenetics, Survival, Recurrence, DNA methylation

## Abstract

**Background:**

HPV-associated HNSCCs have a distinct etiologic mechanism and better prognosis than those with non-HPV associated HNSCCs. However, even within the each group, there is heterogeneity in survival time. Here, we test the hypothesis that specific candidate gene methylation markers (*CCNA1*, *NDN*, *CD1A*, *DCC*, *p16*, *GADD45A*) are associated with tumor recurrence and survival, in a well-characterized, prospective, cohort of 346 HNSCC patients.

**Methods:**

Kaplan-Meier curves were used to estimate survival time distributions. Multivariable Cox Proportional Hazards models were used to test associations between each methylation marker and OST/RPFT after adjusting for known or identified prognostic factors. Stratified Cox models included an interaction term between HPV and methylation marker to test for differences in the associations of the biomarker with OST or RPFT across HPV status.

**Results:**

Methylation markers were differentially associated with patient characteristics. DNA hypermethylation of *NDN* and *CD1A* was found to be significantly associated with overall survival time (OST) in all HNSCC patients (*NDN* hazard ratio (HR): 2.35, 95 % CI: 1.40-3.94; *CD1A* HR: 1.31, 95 % CI: 1.01-1.71). Stratification by HPV status revealed hypermethylation of *CD1A* was associated with better OST and recurrence/persistence-free time (RPFT) (OST HR: 3.34, 95 % CI: 1.88-5.93; RPFT HR: 2.06, 95 % CI: 1.21-3.49), while hypomethylation of *CCNA1* was associated with increased RPFT in HPV (+) patients only (HR: 0.31, 95 % CI: 0.13-0.74).

**Conclusions:**

This study is the first to describe novel epigenetic alterations associated with survival in an unselected, prospectively collected, consecutive cohort of patients with HNSCC. DNA hypermethylation of *NDN* and *CD1A* was found to be significantly associated with increased overall survival time in all HNSCC patients. However, stratification by the important prognostic factor of HPV status revealed the immune marker, *CD1A*, and the cell cycle regulator, *CCNA1* to be associated with prognosis in HPV (+) patients, specifically. Here, we identified novel methylation markers and specific, epigenetic molecular differences associated with HPV status, which warrant further investigation.

**Electronic supplementary material:**

The online version of this article (doi:10.1186/s12885-015-1806-8) contains supplementary material, which is available to authorized users.

## Background

Head and neck cancer is the 6^th^ most common cancer in the world with approximately 600,000 new cases each year and at least 90 % being squamous cell carcinomas [1, 2]. Heavy tobacco and alcohol use are well established risk factors, but high-risk human papillomavirus (HPV) infection has recently been identified as an independent etiologic factor for a subset of head and neck squamous cell carcinomas (HNSCCs) [3]. The overall 5-year survival rate for HNSCC has remained at 50-60 % for the past several decades, primarily due to locoregional or distant metastatic recurrence, which develop in 35- 55 % of patients within two years [4, 5]. Low survival rates are partially due to the fact that almost 60 % of patients are diagnosed after the disease has advanced locally, but also due to pathological, clinical and epidemiological heterogeneity [6, 7] and frequent association of significant co-morbidities.

The incidence of HPV-associated HNSCC has steadily increased, especially in younger patients, while incidence of non-HPV associated HNSCC has declined in recent years [8–10]. HPV-associated HNSCCs have a unique risk profile, a distinct etiologic mechanism, and better prognosis than non-HPV associated HNSCCs [11–14]. HPV (+) patients tend to have cancers almost exclusively located in the oropharynx, be younger with a higher socioeconomic status, and have a less profound use of alcohol and tobacco [13, 15, 16]. Studies show a 60-80 % reduction in mortality in HPV (+) patients compared to patients with non-HPV associated HNSCC [3, 17, 18] regardless of treatment modality or tumor stage. Within each group, however, there is heterogeneity in survival time, with up to 20 % of HNSCCs progressing with distant metastases. Thus, there is strong interest in identifying prognostic markers for both HPV (+) and HPV (-) patients with HNSCC.

Gene-specific DNA methylation has been increasingly recognized as a contributor to the molecular heterogeneity of HNSCC [19, 20]. Several markers have been proposed as biomarkers of prognosis and/or diagnosis [19, 21]. However, there is a need to determine the validity of epigenetic markers considering the divergent etiologic mechanisms. In addition, the extent to which epidemiologic characteristics contribute to the prognostic advantage of HPV (+) tumors is unclear. Combining methylation information with clinical characteristics known to affect survival is crucial to understanding the differences in survival rates by these characteristics and how they may be targeted for intervention.

Here we test the hypothesis that specific candidate gene methylation markers (*CCNA1*, *NDN*, *CD1A*, *DCC*, *GADD45a*, and *p16*) are associated with tumor recurrence and survival, in a well-characterized, prospective cohort of HNSCC patients with extensive epidemiologic, clinical and outcome information, who were treated by a single group of clinicians with a homogenous treatment approach. This approach allows careful consideration of the epigenetic biomarkers in the context of epidemiologic and clinicopathologic characteristics that influence overall and recurrence-free survival.

## Methods

### Recruitment

The University of Michigan’s Head and Neck Specialized Program of Research Excellence (SPORE) approaches every incident, previously untreated HNSCC patient to participate in longitudinal epidemiology studies. This unselected study population represents 28 % of incident HNSCC cases in the state of Michigan. From November 2008 through June 2012, subjects were screened for eligibility and 92 % (n = 513) of subjects approached signed a written, informed consent. Consented subjects completed a baseline questionnaire of demographics, epidemiologic characteristics, and behavior modules. Comorbidity data were abstracted from the medical record and graded by severity (none, mild, moderate, severe) using the Adult Comorbidity Evaluation of 27 conditions organized by 12 systems (ACE-27). Research assistants collected formalin-fixed, paraffin-embedded (FFPE) HNSCC tissue blocks and detailed pathophysiological and clinical data annually until death or the patient was lost to follow-up. This study was approved by the Institutional Review Board of the University of Michigan Medical School.

### Tissue acquisition

The FFPE tissue blocks were collected pretreatment from three possible sources: (1) a biopsy obtained from an outside hospital, (2) a biopsy performed at the University of Michigan hospital, and/or (3) from surgery performed at the University of Michigan. Tissue acquired from the three sources yielded at least one sample for 88 % (n = 450) of the subjects.

### Study population

An expert head and neck pathologist (JM) confirmed tumor histology and screened representative blocks for areas of >70 % cellularity and minimal necrosis. Seventy-two percent (n = 369) of all subjects had sufficient tissue and DNA to yield methylation results. Of these, 15 subjects were excluded for tumors arising from rare sites or non-squamous histology (e.g., unknown primary, nasopharynx, salivary gland, sinus) and 7 subjects were excluded for indeterminate HPV status. One subject was lost to follow up. This resulted in a total of 346 subjects used in the methylation analyses, representing 67 % of the 513 eligible participants screened.

### Follow-up

All patients were followed prospectively at designated intervals by clinicians at the University of Michigan or through contact with referring physicians. The median follow-up period was 27 months for survival and 24 months for recurrence (range: 1-54 months). Number of patients alive and followed for OS at 1, 2 and 3 years were 307, 90 and 85 patients, respectively. Number of patients alive and followed for RPFT at 1, 2 and 3 years were 242, 129 and 53 patients, respectively. Deaths were captured through the Social Security Death Index, yearly survey updates, notification from family, and medical record reviews. Survival time and events were censored as of 4/30/13. Recurrence and persistent disease events were confirmed updated annually during a chart review at every subject’s yearly anniversary of their date of initial diagnosis.

### Target gene selection

Our group recently completed a discovery-based study designed to identify novel prognostic epigenetic biomarkers for patients with HNSCC [22, 23]. *CCNA1* (cyclin A1) was chosen for further testing based on its potential for clinical relevance and the discovery analysis that identified regions of their promoters to be significantly differentially methylated in head and neck cancer patients by HPV status [22]. *NDN* (necdin) and *CD1a* (cluster of differentiation 1a) were also differentially methylated in this discovery analysis, however they were not significant, potentially due to small sample size. *NDN* is an imprinted gene previously implicated in epithelial ovarian, bladder, breast, colorectal, and urothelial cancers, as well as premalignant lesions such as vulval intraepithelial neoplasia and Barrett’s oesophagus, although has not been studied in the context of HNSCC [22–29]. *CD1A* was the first immune gene found to be differentially methylated in the discovery analysis. *CD1A* methylation has not been previously studied in HNSCC, however significant hypermethylation of *CD1B*, *CD1C*, *CD1D* and *CD1E* has been found in HPV (+) HNSCC tumors compared to HPV(-) tumors [30]. *DCC* (deleted in colorectal carcinoma), *GADD45* (growth arrest and DNA damage 45) and *p16* (cyclin-dependent kinase inhibitor) were all previously found to be hypermethylated in HNSCC and were chosen for their role as tumor suppressors and potential involvement with HPV [9, 31–34].

### Microdissection/DNA extraction/bisulfite conversion

Designated areas of FFPE tissue were microdissected from unstained slides and DNA was extracted using the QIAamp DNA FFPE Tissue Kit (Qiagen, Valencia, CA) according to the manufacturer’s protocol. DNA concentration and purity was measured with a NanoDrop spectrophotometer (Thermo Scientific, Waltham, MA). Sodium bisulfite treatment was performed on 250 ng of DNA using the Epitect Bisulfite Kit (Qiagen, Valencia, CA) according to the manufacturer’s recommended protocol.

### HPV testing

HPV status was determined by an ultrasensitive method using real-time competitive polymerase chain reaction (PCR) and matrix-assisted laser desorption/ionization mass spectroscopy, as described in Tang et al. [35]. Multiplex PCR amplification of the E6 region of 15 discrete high risk HPV types (HPV 16, 18, 31, 33, 35, 39, 45, 51, 52, 56, 58, 59, 66, 68 and 73), and human GAPDH control included a competitor oligo identical to each natural amplicon except for a single nucleotide difference. Probes that identify unique sequences in the oncogenic E6 region of each type were used in multiplex single base extension reactions extending at the single base difference between wild-type and competitor HPV so that each HPV type and its competitor were distinguished by mass when analyzed on the MALDI-TOF mass spectrometer as described previously [32, 36–39].

### Methylation analysis

Methylation assays for promoter regions of *DCC, CD1A, GADD45 and NDN*, were designed using PyroMark Assay Design 2.0 software across 5, 2, 5 and 3 CpG sites, respectively (Qiagen, Valencia, CA). The methylation assay for *p16* was adapted from Shaw, et al. and covered 4 CpG sites [40]. The promoter region of *CCNA1* was sequenced using the Sequenom EpiTyper, a MALDI-TOF mass spectrometry based platform across 4 CpG sites. Primer sets are shown in Additional file 1: Table S1. Location of each CpG site and distance to transcription start site are denoted in Additional file 1: Table S2. Bisulfite singleplex PCR amplification was performed using FastStart Taq Polymerase (Roche Diagnostics, Indiana, US) for *CCNA1*, and HotStar Taq® Master Mix Kit (Qiagen Valencia, CA) for all other genes, with a forward and reverse primer concentration of 0.2 mM and 30 ng of bisulfite-converted DNA. Confirmation of PCR product quality and absence of contamination was established from 2 % agarose gels with ethidium bromide staining. Fifteen microliters of each PCR product was combined with the respective sequencing primer and methylation analysis by pyrosequencing was conducted using the Pyromark™ MD System (Biotage) according to manufacturer’s protocol, including single strand binding protein (PyroGold reagents). Four bisulfite and four pyrosequencing controls were generated by mixing unmethylated and methylated control DNA (genomic: EpigenDX; bisulfite-converted: Epitect) to obtain controls with 0 %, 30 %, 60 % and 100 % methylation. Each sample plate was run with all controls. If methylation values of controls were incorrect, all samples on plate were re-run. Measurement of all samples for every methylation marker selected was not possible if there was insufficient quantity of total extracted DNA. Average methylation across all CpG sites measured for each gene were used in all statistical analyses as strong associations between CpG sites of each gene has been previously shown [41].

### Statistical analysis

Overall survival time (OST) and recurrence/persistence-free time (RPFT) were calculated beginning at date of diagnosis. An OST event was defined as death from any cause. For RPFT, an event was defined as any recurrence (local, regional or distant) of the tumor or persistence of the tumor after definitive treatment. In the case of persistence, an RPFT of 1 day after diagnosis was assigned. In the case of death prior to recurrence, a subject was censored for RPFT at the last known date recurrence-free.

Univariate analyses, including Kruskal-Wallis and Wilcoxon-rank tests, were conducted to test for differences in methylation of each gene by clinico-pathological and epidemiological characteristics. The Kaplan-Meier method was employed to estimate survival time distributions and graphically visualize time-to-event outcomes for overall survival time (OST) and recurrence/persistence-free time (RPFT) by methylation. Methylation of each marker was categorized into quartiles for the Kaplan-Meier curves, with quartile 1 containing the lowest values and quartile 4 containing the highest (Additional file 1: Table S3). Statistical differences in curves were tested using the log-rank test.

Multivariable Cox proportional hazard models were used to test associations between each methylation marker and OST/RPFT after adjusting for known or identified prognostic factors. All mean methylation values were log-transformed after adding an offset value of 1. For each outcome, a model with only clinical predictors was developed using a backward selection algorithm (alpha criteria = 0.05) to arrive at a parsimonious model, with the stipulation that stage and disease site would remain in the model regardless of their significance. Variables introduced for potential inclusion were: age, HPV status, ACE comorbidity score (none, mild, moderate, severe), tobacco use (never, former, current within 12 months), and alcohol use (never, former, current within 12 months). The final clinical model for both OST and RPFT included age, tumor stage, disease site, and HPV; the final clinical model for OST included comorbidity score in addition. After data exploration, significant violation to the proportional hazards assumption was observed for HPV. Stratified Cox proportional hazard models were performed that allowed differing baseline hazard functions for HPV+ and HPV- groups accounting for the non-proportional hazards observed in our data. These stratified Cox proportional hazards models included the same adjustment covariates as the unstratified version, and included an additional interaction term between HPV and methylation marker to test for differences in the associations of the biomarker with OST or RPFT across HPV status. Finally, each methylation marker was added to both the stratified and un-stratified version of the clinical Cox models to assess associations between the marker and outcome after covariate adjustment. All methylation measurements were standardized to interquartile ranges (IQR) of each respective marker (Additional file 1: Table S3). Therefore, hazard ratios (HRs) are interpreted as a comparison between those with methylation in the 25th percentile compared to those with methylation in the 75th percentile.

Unadjusted p-values are presented, however authors advise that a significance finding near the threshold of p < 0.05 should be interpreted with caution. Due to multiple tests being performed (each outcome was modeled for 6 genes), a more conservative Bonferroni threshold for significance was calculated as p < 0.004 (0.05/12) and reflected in superscripts in the Cox model results (Table 4).

Statistical analyses were conducted in R 3.1.1 and SAS 9.3.

## Results

### HNSCC patient characteristics

The mean age of the HNSCC patients was 59.7 years and consisted of 75 % males (Table 1). Cancer sites were mostly oropharyngeal and oral cavity (36 % each) while laryngeal cancers made up about 24 % of cases and only 3 % of cases were hypopharyngeal. Sixty-one percent of cases were stage IV. Forty-six percent of patients had mild comorbidity status, while 26 % had moderate and 8 % had severe comorbidity. Forty-two percent of patients were classified as current smokers, or having quit within the past 12 months, while 36 % were former smokers (quit more than one year ago) and 22 % were never smokers. Distributions of all patient characteristics are listed in Table 1.

**Table 1:** Patient clinical and epidemiological characteristics, N = 346

Characteristic		N	(%)	Mean (std), range
Age at Dx (years)				59.7 (11.5), 25-93
Gender	Male	259	75 %	
	Female	87	25 %	
Cancer Site	Larynx/Glottic	83	24 %	
	Oral Cavity	126	36 %	
	Oropharynx	125	36 %	
	Hypopharynx	12	3 %	
Cancer Stage	I/Cis	47	14 %	
	II	35	10 %	
	III	52	15 %	
	IV	212	61 %	
Comorbidities (ACE)	None	91	26 %	
	Mild	158	46 %	
	Moderate	70	20 %	
	Severe	27	8 %	
HPV status	Positive	135	39 %	
	Negative	211	61 %	
Tobacco Use	Current (within past 12 months)	145	42 %	
	Former (quit > 12 months)	125	36 %	
	Never	76	22 %	
Pack Years, n = 257	(cigs only)			35.6 (30.0), 0.1-171.0
Pack Years, n = 264	(sum of cigs, cigars, pipe)			37.7 (33.3), 0.07-242.9
Alcohol Use, n = 244	Never	29	8 %	
	Former (quit >12 months)	227	66 %	
	Current (within past 12 months)	90	26 %	
Treatment, n = 335	Surgery alone	67	20 %	
	Radiation alone	34	10 %	
	Surgery + Radiation	34	10 %	
	Radiation + Chemotherapy	138	41 %	
	Surgery + Radiation + Chemotherapy	39	12 %	
	No Treatment prior to death	23	7 %	
Persistent Disease^a^		29	8 %	
Median Follow-up for Survival		27 months		
Median Follow-up for Recurrence/Persistence		24 months		
Recurrence/Persistence^b^		81		
Death		78		
KM estimate 2 year RPFT^c^			75 %	
KM estimate 2 year OST^d^			79 %	

^a^Disease considered persistent if patient never became disease free. ^b^Recurrence of the HNSCC in the primary, regional and/or a distant location. Persistent patients whose disease never cleared after treatment are included and considered recurrent with a recurrence time = 1 day. ^c^*RPFT* Recurrence/Persistent Free Time defined as time from diagnosis to recurrence event or end of follow-up. End of follow-up is the last date where patient was reviewed for recurrence. Patients whose disease never cleared after treatment are considered recurrent with a recurrence time = 1 day. ^d^*OST* Overall Survival Time. Death from any cause considered an event. Overall survival time defined from date of diagnosis by UM physician

Patients with HPV (+) tumors (n = 135) were on average younger than patients with HPV (-) tumors (n = 211) (mean age = 57 years, SD: 9.6 years for HPV (+) patients and mean age = 61.4 years, SD: 12.3 years for HPV (-) patients, *p*-value = 0.0002) (Table 2). The majority of HPV (-) patients had cancers of the oral cavity (52 %) whereas the majority of HPV (+) patients had cancer of the oropharynx (OP) (78 %). HPV was detected in 22 % of non-oropharyngeal (non OP) sites. Comparisons between HPV (+) patients presenting in OP and non-OP sites revealed similar 2-year overall survival times (Kaplan Meier Estimate (KM) (95 % CI): 92 % (87 %, 98 %); 89 % (77 %, 100 %); respectively, *p*-value = 0.71) as well as similar recurrence-free survival times (KM (95 % CI): 85 % (78 %, 93 %); 80 % (66 %, 98 %), respectively, *p*-value = 0.59; Additional file 1: Figure S1) More patients with HPV (+) tumors were diagnosed with stage IV tumors than those patients with HPV (-) tumors (77 % vs 51 %) primarily due to a higher frequency of patients with N2 neck disease that is common in HPV related cancers [42, 43]. Most HPV (-) patients were current smokers (48 %) while HPV (+) patients had lower proportions of current (32 %) smokers and similar frequencies of former (37 %) and never (31 %) smokers. Pack-years of cigarettes only use was higher in HPV (-) patients (mean: 38.4 pack-years) compared to HPV (+) patients (29.9 pack-years, *p*-value = 0.02). Distributions of patient characteristics by HPV status are listed in Table 2.

**Table 2:** Patient clinical and epidemiological characteristics by HPV status, N = 346

		HPV (–)		HPV (+)		HPV (-)	HPV (+)	
Characteristic		N	(%) among HPV(-)	N	(%) among HPV(+)	Mean (SD)	Mean (SD)	*p*-value
Age at Dx (years)		211		135		61.4 (12.3)	57.0 (9.6)	0.0002
Gender	Male	139	66 %	120	89 %			<0.0001
	Female	72	34 %	15	11 %			
Cancer Site	Larynx/Glottic	72	34 %	11	8 %			<0.0001
	Oral Cavity	110	52 %	16	12 %			
	Oropharynx	20	9 %	105	78 %			
	Hypopharynx	9	4 %	3	2 %			
Cancer Stage	I/Cis	36	17 %	11	8 %			<0.0001
	II	28	13 %	7	5 %			
	III	39	18 %	13	10 %			
	IV	108	51 %	104	77 %			
Comorbidities (ACE)	None	41	19 %	50	37 %			0.001
	Mild	99	47 %	59	44 %			
	Moderate	50	24 %	20	15 %			
	Severe	21	10 %	6	4 %			
Tobacco Use	Current (within past 12 months)	102	48 %	43	32 %			0.001
	Former (quit > 12 months)	75	36 %	50	37 %			
	Never	34	16 %	42	31 %			
Pack Years, n = 257	(cigs only)					38.4 (25.6)	29.9 (28.8)	0.02
Pack Years, n = 264	(cigs, cigars, pipe)					41.8 (34.5)	29.6 (29.4)	0.005
Alcohol Use, n = 244	Current (within past 12 months)	63	30 %	27	20 %			0.01
	Former (quit > 12 months)	126	60 %	101	75 %			
	Never	22	10 %	7	5 %			
Treatment, n = 335	Surg alone	53	26 %	14	11 %			<0.0001
	Rad alone	23	11 %	11	8 %			
	Surg + Rad	30	15 %	4	3 %			
	Rad + Chemo	45	22 %	93	70 %			
	Surg + Rad + Chemo	29	14 %	10	8 %			
	No Treatment prior to death	22	11 %	1	1 %			
Post Treatment Status	Free of Disease	186	88 %	131	97 %			0.004
	Persistent Disease	25	12 %	4	3 %			
KM estimate 2 year RPFT			69 %		84 %			0.001
KM estimate 2 year OST			71 %		91 %			<0.0001

-Percentages will not add to 100 % horizontally. % presented is proportion of clinical or epidemiological subgroup among HPV status category. -*p*-values calculated by Wilcoxon-rank tests for continuous variables and Kruskal-Wallis tests for categorical variables. Log-rank test used for KM analysis

### Tumor methylation differs by epidemiologic characteristics

Methylation of *CD1A* differed across clinically relevant age groups, decreasing with increasing age (Table 3). HPV status was significantly associated with several markers, as expected. Methylation of *CCNA1*, *NDN*, *CD1A*, and *DCC* was higher, while methylation of *p16*was lower, in HPV (+) tumors compared to HPV (-) tumors. Increasing number of total pack-years across all tobacco types was significantly associated with decreased methylation of *NDN* and *CD1A*. Tobacco use (current, former, never user) was also considered separately as these data were complete and more reliable than pack-years for most patients (Table 3). Tobacco use was significantly associated with methylation of *NDN*, *CD1A*, and *DCC* (*p-value*  = 0.02; *p-value*  < 0.001; *p-value*  = 0.03, respectively). Interestingly, former smokers had lower methylation of these genes compared to never or current smokers. Trends of each methylation marker differed significantly across disease site. Methylation of *NDN* was higher in patients with higher stage tumors (*p -value* < 0.001), while a trend for *CCNA1* methylation with stage was U-shaped, with higher methylation at stages I and IV (*p -value* = 0.01). As comorbidity status increased, methylation of *CD1A* increased (*p-value* = 0.006). Finally, methylation of *NDN* and *DCC* was significantly higher in males compared to females (*p-value* =0.003; *p-value* =0.002, respectively).

**Table 3:** Methylation markers by patient characteristics

	CCNA1		NDN		CD1A		DCC		p16		GADD45	
	median (range)	*p*	median (range)	*p*	median (range)	*p*	median (range)	*p*	median (range)	*p*	median (range)	*p*
Age												
<40 years	19.5 (14.5, 64.7)	0.4	40.8 (34.5, 72.8)	0.4	78.6 (56.8, 91.2))	0.02	21.3 (9.9, 62.3)	0.4	3.1 (0.6, 25.0)	0.5	1.5 (1.0, 2.8)	0.5
40-60	25.8 (3.0, 90.5)		44.6 (10.8, 84.2)		71.0 (22.2, 93.9)		33.2 (3.1, 85.8)		2.3 (0, 70.1)		1.4 (0, 3.9)	
≥60	27.3 (5.5, 77.0)		43.9 (11.2, 84.9)		68.6 (21.4, 90.9)		33.7 (3.0, 79.8)		2.2 (0, 59.8)		1.4 (0, 16.0)	
HPV												
Positive	33.3 (3.0, 76.0)	<0.001	52.7 (14.0, 84.9)	<0.001	77.6 (25.1, 93.9)	<0.001	44.7 (3.1, 85.8)	<0.001	1.9 (0, 59.8)	<0.001	1.4 (0.4, 3.9)	0.8
Negative	20.9 (5.5, 90.5)		40.0 (10.8, 80.3)		65.4 (21.4 (92.2)		26.0 (3.0, 83.3)		2.5 (0, 70.1)		1.4 (0, 16)	
Pack Years												
0	26.6 (3.0, 90.5)	0.2	46.4 (14.0, 84.2)	0.01	79.7 (47.2, 93.9)	<0.001	36.7 (3.1, 79.8)	0.1	2.2 (0, 26.9)	0.7	1.5 (0.3, 3.7)	0.2
0-10	31.5 (13.3, 63.3)		45.9 (24.6, 75.7)		70.5 (32.4, 91.1)		40.5 (10.8, 71.7)		2.1 (0, 46.6)		1.4 (0.4, 16.0)	
10-20	23.6 (6.8, 64.8)		46.4 (10.8, 76.8)		73.0 (26.7, 91.7)		32.7 (6.1, 75.1)		2.2 (0, 70.1)		1.6 (0.7, 3.9)	
20+	25.5 (6.3, 77.0)		41.3 (11.2, 84.9)		64.2 (21.4, 92.0)		31.4 (3.0, 85.8)		2.4 (0, 59.8)		1.4 (0, 3.9)	
Tobacco												
Current	25.5 (6.3, 76.0)	0.8	45.0 (10.8, 84.9)	0.02	69.5 (25.1, 91.7)	<0.001	39.5 (4.2, 83.3)	0.03	2.2 (0, 70.1)	0.7	1.4 (0, 16)	0.5
Former	25.6 (6.8, 77.0)		42.2 (11.2, 80.3)		64.4 (21.4, 90.0)		29.3 (3.0, 85.8)		2.4 (0, 46.6)		1.4 (0, 3.9)	
Never	26.6 (3, 90.5)		46.4 (14.0, 84.2)		79.7 (47.2, 93.9)		35.7 (3.1, 79.8)		2.2 (0, 26.9)		1.5 (0.3, 3.7)	
Site												
Larynx/Glottic	24.5 (8.0, 90.5)	<0.001	41.2 (11.2, 84.9)	<0.001	56.2 (21.6, 92.2)	<0.001	26.3 (3.1, 83.3)	<0.001	2.4 (0, 51.8)	<0.001	1.4 (0, 3.9)	0.02
Oral Cavity	22.0 (5.5, 77.0)		40.0 (10.8, 62.8)		70.6 (21.4, 90.2)		28.4 (3.0, 67.2)		2.6 (0, 70.1)		1.4 (0, 16.0)	
Oropharynx	32.3 (3.0, 76.0)		52.7 (21.8, 84.2)		77.5 (21.6, 93.9)		42.5 (6.1, 81.6)		2.0 (0, 39.9)		1.4 (0.4, 3.9)	
Hypopharynx	15.6 (9.0, 64.0)		44.2 (17.2, 91.0)		60.5 (25.1, 90.6)		54.5 (12.3, 85.8)		2.6 90, 59.8)		1.1 (0, 1.6)	
Stage												
I/Cis	27.5 (11.8, 77.0)	0.01	38.6 (11.2, 80.3)	<0.001	68.1 (30.3, 89.4)	0.8	26.7 (3.0, 67.2)	0.1	2.5 (0, 39.0)	0.1	1.3 (0, 3.5)	0.3
II	22.4 (12, 74.3)		41.2 (17.2, 84.2)		68.8 (21.6, 88.3)		40.8 (7.9, 83.3)		2.3 (0, 59.8)		1.4 (0.6, 4.6)	
III	21.3 (6.8, 61.3)		40.4 (15.3, 69.7)		71.2 (21.4, 90.2)		28.8 (3.9, 75.1)		2.6 (0, 70.1)		1.5 (0, 16.0)	
IV	27.6 (3.0, 90.5)		46.8 (10.8, 84.9)		70.5 (21.6, 93.9)		35.9 (3.1, 85.8)		2.2 (0, 51.8)		1.4 (0, 3.9)	
Comorbidities												
None	29.5 (6.3, 74.3)	0.4	46.0 (20.2, 84.2)	0.3	74.8 (32.4, 93.9)	0.006	33.1 (5.6, 75.2)	0.6	2.5 (0, 39.9)	0.4	1.5 (0.4, 4.6)	0.1
Mild	25.5 (3.0, 77.0)		43.3 (10.8, 84.9)		69.5 (21.6, 92.0)		35.4 (3.0, 85.8)		2.2 (0, 70.1)		1.4 (0, 16.0)	
Moderate	23.0 (6.8, 90.5)		44.0 (15.0, 79.3)		69.2 (21.4, 92.2)		29.8 (3.5, 79.8)		2.2 (0, 59.8)		1.3 (0, 3.9)	
Severe	30.5 (13.5,63.3)		44.6 (11.2, 80.3)		65.4 (21.6, 85.1)		43.0 (6.2, 83.3)		1.8 (0, 51.8)		1.2 (0, 3.9)	
Gender												
Male	26.5 (3.0, 90.5)	0.2	45.6 (13.0, 84.9)	0.003	69.9 (21.4, 93.9)	0.4	36.1 (3.1, 85.8)	0.002	2.3 (0, 70.1)	0.9	1.4 (0, 3.9)	0.5
Female	23.8 (6.3, 77.0)		40.5 (10.8, 68.9)		69.5 (21.6, 91.7)		25.4 (3.0, 75.1)		2.1 (0, 46.6)		1.4 (0, 16.0)	

Distribution of methylation markers across patient characteristics. *p*-values calculated by Kruskal-Wallis tests. Methylation values are expressed as percentages

Stratification by HPV status across these epidemiologic and epigenetic characteristics revealed HPV (+) tumors were hypermethylated across *CCNA1*, *NDN*, *CD1A*, and *DCC*, but hypomethylated in *p16* as compared to HPV(-) tumors, regardless of clinical epidemiological traits (data not shown).

### Tumor methylation and survival/recurrence

Kaplan-Meier curves for the association between methylation, categorized into quartiles, and OST and RPFT across all HNSCC patients, are shown in Additional file 1: Figure S2. Differences in methylation of both *CD1A* and *NDN* were significantly associated with OST in all HNSCC patients (*p-value* = 0.005; *p-value* = 0.001, respectively). The lowest quartile of methylation of *CD1A* (Q1) was associated with the lowest probability of OST (0.68 at 24 months) whereas higher methylation in Q2, Q3 and Q4 clustered above a probability of OST of 0.80 at 24 months. Methylation of the lowest quartile of *NDN* was associated with the lowest probability of OST. In terms of RPFT, there were significant associations with *CCNA1*, *DCC* and *NDN* methylation in all HNSCC patients. *NDN* revealed a trend of increasing probability of RPFT with increasing quartile of methylation. The lowest and highest quartiles of *CCNA1* and *DCC* had the lowest probabilities of RPFT.

When patients were separated and analyzed by HPV status, distinct associations between methylation markers and OST/RPFT were identified (Additional file 1: Figures S3 and S4). In HPV (+) patients, higher quartiles of methylation of *CD1A* in Q3 and Q4 were associated with better OST compared to the lower quartiles (*p-value* = 0.03). Within HPV (-) patients, *NDN* and *DCC* were significantly associated with probability of OST. Low methylation of *NDN* in Q1 had the lowest probability of OST compared to methylation in all other quartiles (*p -value* = 0.04). *DCC* methylation in Q2 was associated with the highest probability of OST (*p -value* = 0.005). A similar pattern was observed for *DCC* methylation and RPFT, although with *DCC*, it was Q1 had much poorer probability of recurrence/persistence compared to other quartiles (*p-value* = 0.002). (Additional file 1: Figure S4)

### NDN and CD1A methylation are novel markers of survival in HNSCC patients

Multivariable Cox proportional hazards models adjusting for site, stage, HPV status, age and comorbidity score showed significant associations of *NDN* and *CD1A* gene promoter methylation with survival in all HNSCC patients (Table 4). Treatment was not included in any models as it was confounded by site. Those with *NDN* methylation in the 25^th^ percentile had a 2.4 times higher hazard of a death event compared to those with methylation in the 75^th^ percentile (95 % CI: 1.40-3.94). Patients with *CD1A* methylation in the 25^th^ percentile had a 1.3 times higher hazard of a death event compared to those with methylation in the 75^th^ percentile upon adjustment (95 % CI: 1.01-1.71). These results indicate that for all HNSCC patients, hypermethylation of *NDN* and *CD1A* are associated with better patient survival.

**Table 4:** Stratified Cox Proportional Hazards Analysis comparing overall survival time (OST) and recurrence/persistence free time (RPFT) by gene methylation

			Multivariable cox model	Stratified cox proportional hazards model		
	Gene methylation^a^	N^b^	Multivariable HR (95 % CI)^c,d^	Stratified model among HPV + ^c,e^	Stratified model among HPV-^c,e^	*p*-value^f^
OST	*CCNA1*	341	1.13 (0.81, 1.59)	0.62 (0.25, 1.52)	1.25 (0.86, 1.81)	0.16
	*NDN*	346	2.35 (1.40, 3.94) **	3.12 (0.95, 10.21)	2.22 (1.25, 3.92)*	0.61
	*CD1A*	342	1.31 (1.01, 1.71)*	3.34 (1.88, 5.93)**	1.13 (0.84, 1.51)	0.001**
	*DCC*	344	1.30 (0.95, 1.78)	1.45 (0.59, 3.60)	1.28 (0.91, 1.79)	0.79
	*p16*	343	1.11 (0.93, 1.32)	0.76 (0.48, 1.19)	1.17 (0.97, 1.42)	0.08
	*GADD45*	340	0.88 (0.66, 1.17)	0.80 (0.43, 1.50)	0.91 (0.66, 1.25)	0.73
RPFT	*CCNA1*	334	0.81 (0.57, 1.14)	0.31 (0.13, 0.74)*	0.96 (0.66, 1.42)	0.02*
	*NDN*	339	1.86 (1.12, 3.07)*	2.08 (0.70, 6.19)	1.77 (1.00, 3.13)	0.79
	*CD1A*	335	1.24 (0.95, 1.62)	2.06 (1.21, 3.49)*	1.07 (0.79, 1.46)	0.04*
	*DCC*	337	1.32 (0.96, 1.83)	0.97 (0.40, 2.34)	1.37 (0.97, 1.94)	0.47
	*p16*	336	1.00 (0.84, 1.19)	0.91 (0.57, 1.46)	1.00 (0.84, 1.20)	0.72
	*GADD45*	333	1.12 (0.87, 1.46)	0.84 (0.49, 1.42)	1.23 (0.91, 1.66)	0.22

Each row represents the hazard ratios in the multivariable or stratified Cox Proportional Hazards model for each gene. The top panel lists all overall survival time models and the bottom panel lists all recurrence/persistence-free survival time models. ^a^All genes log-transformed to better fit the linear functional relationship between predictor and outcome ^b^*n* number of subjects in models ^c^All methylation measurements standardized to interquartile ranges. HRs are interpreted as comparison between those with methylation in the 25th percentile compared to those with methylation in the 75th percentile ^d^Multivariable HR calculated from a multivariable Cox Proportional Hazards model after controlling for: age, HPV status, disease site, stage, and comorbidity score in the OST model and age, HPV status, disease site and stage in the RPFT model. ^e^Multivariable HR (95 % CI) calculated from a stratified multivariable Cox Proportional Hazards model to allow for differing baseline hazard functions for HPV+ and HPV- groups. These models include an interaction of HPV status and gene methylation. Hazards listed for each HPV group in the stratified models calculated from this interaction term. Stratified OST models control for: age, disease site, stage, and comorbidity score and stratified RPFT models control for: age, disease site and stage. ^f^*p*-value for interaction term. *p-value < 0.05; **significance at the Bonferroni-adjusted *p*-value < 0.004

To determine the extent to which HPV status may have played a role in these findings, stratified Cox models with interaction between methylation level and HPV status were used to measure associations separately within each group (Table 4). Divergent associations were found in patients based on their HPV status. The test for interaction indicated differences in OST for *CD1A* (*p*-value = 0.001) and in RPFT for *CD1A* (*p*-value = 0.04) and *CCNA1* (*p*-value = 0.02). In HPV (+) patients, *CD1A* methylation in the 25^th^ percentile revealed a 3.34 times higher hazard of a death event than those in the 75^th^ percentile (95 % CI: 1.88-5.93). In addition, patients had twice the hazard of a tumor recurrence or persistence if they were at the 25^th^ percentile of *CD1A* methylation compared to the 75^th^ percentile. These results suggest that hypermethylation of *CD1A* is associated with better survival and lower recurrence/persistence in HPV (+) patients specifically. Conversely, for *CCNA1*, HPV (+) patients had a 0.31 times lower hazard of a recurrent event or persistence comparing those in the 25^th^ percentile to those in the 75^th^ percentile of methylation, indicating that hypomethylation of this gene is associated with lower risk of recurrence/persistence in HPV(+) patients.

## Discussion

Changes in methylation patterns are one of the most frequent events in human neoplasms. Epigenetic alterations have been increasingly recognized to play a role in the complex mechanisms of head and neck carcinogenesis [19, 21]. This study is the first to describe novel epigenetic alterations associated with survival in an unselected, prospectively collected, consecutive cohort of patients with HNSCC. Our strongest findings support overall survival from HNSCC to be associated with *NDN* methylation in all patients and *CD1A* methylation in HPV (+) patients. These associations are significant at *p*-value < 0.004, the Bonferroni-adjusted *p*-value for multiple testing. Despite weaker associations, likely due to lower number of patients and events, there are indications that gene methylation is important in recurrence/persistence of HNSCC. Hypermethylation of *NDN* is associated with lower recurrence/persistence overall whereas hypermethylation of *CD1A* and hypomethylation of *CCNA1* is associated with lower recurrence/persistence in HPV (+) patients only. These unique discoveries raise new questions about why these specific epigenetic changes differ among biologically distinct subsets of HNSCC patients (HPV + versus HPV -) and if these differences are linked to HPV status or other factors (e.g. tumor-host immunity, oncogene mutations).

*NDN* is a maternally imprinted gene that has monoallelic expression. It encodes necdin, a protein that interacts with p53 to suppress growth and induce cell cycle arrest [26]. Although necdin is a p53 target gene involved in cell growth arrest and found to be dysregulated in urothelial, prostate, breast, bladder, lung and ovarian cancers, it has only recently been implicated in HNSCC [22, 23, 26–30]. Our adjusted Cox model analyses revealed hypermethylation of *NDN* was associated with better survival in all HNSCC patients. Although the function of *NDN* is unknown, previous literature has implicated necdin to act as a “switch”, promoting quiescence under steady state conditions but bypassing p53 responses to promote proliferation or suppressing p53-induced apoptosis in a stressful state [25, 26, 44]. Our findings support the oncogenic function of this gene within tumor cells in that hypermethylation of this gene is beneficial for patient survival.

*CD1A* encodes an immune protein responsible for presenting antigens by dendritic Langerhans cells to T lymphocytes, such as natural killer cells. Findings from our adjusted Cox model validated the difference seen in Kaplan-Meier survival curves across quartiles of *CD1A* methylation. Our analyses revealed hypermethylation was associated with better overall survival for all HNSCC patients and, for better survival and lower recurrence in HPV (+) patients specifically, even after adjusting for important clinical variables. Recent studies have highlighted important differences in immunologic status in patients’ peripheral blood and tumor microenvironment according to HPV status [45, 46]. Patients with non-HPV related HNSCC tend to be significantly immunosuppressed and this immune suppression is associated with worse outcomes. CD1A dysregulation could negatively impact activation of suppressor and regulatory T cells systemically as well as tumor associated macrophages in the microenvironment, benefiting patients with HPV+ cancers. Further studies of CD1A expression and the specific immune abnormalities associated with HPV status are underway.

Hypomethylation of *CCNA1* was associated with lower hazard of recurrence/persistence in HPV (+) patients only. *CCNA1* is a cell cycle regulator that binds to retinoblastoma, E2F transcription factor and p21 family proteins to promote cell cycle progression. This pathway plays a particularly important regulatory role in cell proliferation with HPV infection. Our results suggest that decreased methylation of this cell cycle regulator is protective against recurrence events. Our findings are consistent with previous findings in HPV (+) HNSCC patients [22, 30, 47, 48]. However, several studies have shown that promoter methylation of *CCNA1* had no effect on protein or gene expression in HPV-positive HNSCC in spite of a strong correlation between *CCNA1* overexpression and HPV positivity, suggesting that HPV may induce both promoter hypermethylation and overexpression [47, 49, 50]. Prognostically, HPV induced cyclin A1 overexpression has been associated with a lower recurrence rate in HNSCC [47].

Considering *CD1A* is an immune regulatory gene and *CCNA1* is involved in response pathways to HPV infection, it is plausible that these genes would play a larger role in more antigenic cancers with a viral etiology. *NDN*, which is involved in cell cycle regulation via p53 pathways, is likely to be important in regulation of altered cell cycle pathways contributing to the process of tumorigenesis [26]. However, it remains unclear to what extent these significant associations with survival are due to treatment efficacy or reflect epigenetic variations due to co-morbidities or health behaviors. In contrast to other reports from large outcome studies, there were no significant differences in survival outcomes with respect to co-morbidities, suggesting that the epigenetic changes more likely were reflective of differences in tumor biology rather than co-morbidity. Larger epigenetic studies of smoking, diet and co-morbidities and associations with known genetic mutations are currently underway.

Our sensitive method of HPV detection has been previously validated with other commonly used assays to detect HPV [39]. As expected, a majority of HPV (+) patients presented in the oropharynx, although our use of this assay allowed us to detect HPV in 22 % of HPV (+) patients with non-oropharynx sites, 16 % of which were in the oral cavity. In the last decade, oral HPV prevalence has increased in HNSCC. Previous studies have reported prevalence of HPV in oral cavity tumors to be between 4-20 % [3, 51–53] . This is likely explained by the significant increase in incidence of HPV-associated oral squamous cell carcinomas (OSCCs) since 2000, compared to the relatively stable incidence trends of non-HPV associated OSCCs [11]. Our cohort is a prospective, unselected patient population that is likely representing the changing epidemiology affecting HPV-associated OSCC incidence. This is further supported by our finding of similar 2-year survival times of HPV (+) patients with OP and non-OP tumors. The low proportion of HPV (+) patients with tumors in the larynx and hypopharynx in our study fell within previously reported figures [54, 55].

Epidemiologic and clinical characteristics have generally been used to understand cancer phenotype, determine prognosis and inform treatment plans for patients. Epidemiologic factors such as smoking history, nutrition and comorbidity are well known significant prognostic factors for overall survival and indicate the importance of including such factors in studies of new molecular markers [8, 56, 57]. In the last decade, the clinical importance of better understanding tumor biology in HNSCC has emerged through validation of HPV status as a significant molecular predictor of patient survival and recurrence. Differences in patient outcomes according to HPV status are so dramatic that many investigators believe they reflect a new and unique phenotype that could justify significant de-intensification of therapy [8, 58]. In this study we have identified novel methylation markers and specific, epigenetic molecular differences within the setting of the generalized hypermethylation phenotype associated with HPV status, which warrant further investigation. The findings support biological implications of epigenetic markers on patient survival and their potential usefulness in identifying unique subsets of patients with varied outcomes. Several markers show expected associations with patient characteristics. Methylation of the immune marker, *CD1A*, is indicative of comorbidity status, while several markers are differentially methylated according to HPV status, validating our previous study [22]. However, the finding of associations of methylation markers with characteristics such as stage, gender and tobacco status is new. Former smokers had lower methylation of *NDN* and *CD1A* compared to never and current smokers. Because total pack years is inversely associated with these genes, perhaps former smokers differ because duration of exposure and/or exposure at an early age is integral in the initiation of processes that permits carcinogenesis.

## Conclusions

Our cohort shows the expected associations established in previous literature, such as the relationships between stage, site and HPV status with overall survival time, and the expected population characteristics of a HNSCC cohort established by previous studies, providing assurance that the new associations with survival demonstrated with this cohort are meaningful and can be extrapolated to the general HNSCC patient population. Identification of significant epigenetic markers of biologic tumor behavior and outcome should open new horizons for investigations and interventions directed at reversible gene alterations and potentially identify novel therapeutic targets.

## Additional file

Additional file 1: **Table S1.** Primer Sets and PCR Conditions for Methylation Analysis. **Table S2.** CpG Locations. **Table S3.** Quartile and Interquartile Ranges. **Figure S1**. Comparison of survival and recurrence outcomes by HPV (+) oropharyngeal (OP) and HPV (+) non-OP patients. Significant difference between groups is determined by a univariable Cox Proportional Hazards model for each outcome with a p-value <0.05. a) Probabilities of overall survival time did not differ for OP HPV (+) patients and non-OP HPV (+) patients (p-value = 0.71). b) Probabilities of recurrence/persistence free time did not differ for OP HPV (+) patients and non-OP HPV (+) patients (p-value = 0.59). **Figure S2.** Kaplan-Meier curves of overall survival time and recurrence-free survival time for each methylation marker divided categorized into quartiles. **Figure S3.** Kaplan- Meier curves of overall survival time for each methylation marker, stratified by HPV status for overall survival time. **Figure S4.** Kaplan- Meier curves of overall survival time for each methylation marker, stratified by HPV status for recurrence-free survival time. (DOCX 22 kb)

## Abbreviations

HPV: Human papilloma virus
HNSCC: Head and neck squamous cell carcinoma
OST: Overall survival time
RPFT: Recurrence/persistence-free time
IQR: Interquartile range
*CCNA1*: Cyclin A1
*NDN*: Necdin
*CD1a*: Cluster of differentiation 1a
*DCC*: Deleted in colorectal carcinoma
*GADD45*: Growth arrest and DNA damage 45
*p16*: Cyclin-dependent kinase inhibitor
