# Supplementary material for: NDN and CD1A are novel prognostic methylation markers in patients with head and neck squamous carcinomas
Source: BMC Cancer. 2015 Oct 30;15:825. doi: 10.1186/s12885-015-1806-8 (PMC4628358; doi:10.1186/s12885-015-1806-8)
Supplement: Additional file 1: — Table S1. Primer Sets and PCR Conditions for Methylation Analysis. Table S2. CpG Locations. Table S3. Quartile and Interquartile Ranges. Figure S1. Comparison of survival and recurrence outcomes by HPV (+) oropharyngeal (OP) and HPV (+) non-OP patients. Significant difference between groups is determined by a univariable Cox Proportional Hazards model for each outcome with a p-value <0.05. a) Probabilities of overall survival time did not differ for OP HPV (+) patients and non-OP HPV (+) patients (p-value = 0.71). b) Probabilities of recurrence/persistence free time did not differ for OP HPV (+) patients and non-OP HPV (+) patients (p-value = 0.59). Figure S2. Kaplan-Meier curves of overall survival time and recurrence-free survival time for each methylation marker divided categorized into quartiles. Figure S3. Kaplan- Meier curves of overall survival time for each methylation marker, stratified by HPV status for overall survival time. Figure S4. Kaplan- Meier curves of overall survival time for each methylation marker, stratified by HPV status for recurrence-free survival time. (DOCX 22 kb) [file 12885_2015_1806_MOESM1_ESM.docx]

***NDN* and *CD1A* are novel prognostic methylation markers and differ by HPV status in patients with head and neck squamous carcinomas**

Virani, S^1^, Bellile E^5^, Bradford CR^2^, Carey TE^2^, Chepeha DB^2^, Colacino JA^1^, Helman JI^6,7^ , McHugh JB^4^, Peterson, LA^2^, Sartor, MA^3^, Taylor JMG^5^, Walline HM^2^, Wolf GT^2^, Rozek LS^1,2*^.

^1^Department of Environmental Health Sciences, University of Michigan School of Public Health, Ann Arbor, Michigan, United States of America

^2^Department of Otolaryngology, University of Michigan Medical School, Ann Arbor, Michigan, United States of America

^3^Department of Computational Medicine and Bioinformatics, University of Michigan, Ann Arbor, Michigan, United States of America

^4^Department of Pathology, University of Michigan Medical School, Ann Arbor, Michigan, United States of America,

^5^Department of Biostatistics, University of Michigan, School of Public Health, Ann Arbor, Michigan, United States of America

^6^Department of Oral-Maxillofacial Surgery, University of Michigan Dental School, Ann Arbor, Michigan, United States of America

^7^Department of Surgery, University of Michigan Medical School, Ann Arbor, Michigan, United States of America

* Correspondence/reprints addressed to:

Laura Rozek

1415 Washington Heights

Environmental Health Sciences 6630 SPH

Ann Arbor, MI USA 48109-2029

(734) 615-9816

| Name | Email |
| --- | --- |
| Shama Virani | shamav@umich.edu |
| Emily Bellile | lighte@med.umich.edu |
| Carol R Bradford | cbradfor@umich.edu |
| Thomas E Carey | careyte@med.umich.edu |
| Douglas B Chepeha | douglas.chepeha@uhn.ca |
| Justin A Colacino | colacino@umich.edu |
| Joseph I Helman | jihelman@umich.edu |
| Jonathan B McHugh | jonamch@med.umich.edu |
| Lisa A Peterson | peterlis@med.umich.edu |
| Maureen A Sartor | sartorma@umich.edu |
| Jeremy MG Taylor | jmgt@umich.edu |
| Heather M Walline | hwalline@umich.edu |
| Greg T Wolf | Gregwolf@med.umich.edu |
| Laura S Rozek | rozekl@umich.edu |

.

**Supplemental Tables**

For transparency, we have included our primer sequences in a supplemental table. We have also included the precise locations of CpG sites measured in each gene promoter region for clarity

Table 1S. Primer Sets and PCR Conditions for Methylation Analysis

| **Gene** | **Forward (5’-3’)** | **Reverse (5’-biotin-3’)** | **Sequencing (5’-3’)** | **Annealing Temperature** *(°C) / #cycles* |
| --- | --- | --- | --- | --- |
| *CCNA1* | GGTTGGTTATTAGAGGGTGATTTTTTATTGGGG | CAGTAATACGACTCACTATAGGGAGAA-GGCTAAAAAAACATTCTAACAAACCTCCA |  | 48 / 40 |
| *DCC* | GGTTGGTTGATTAGGATTGTTGTAATT | CCCCTCACTATACCCCAATACCCATCTA | TTGATTAGGATTGTTGTAATTT | 52 / 45 |
| *NDN* | TTTTTTAGAAATTTTAGGGTTGTTGTGTAT | AACCCAAAAACCCTACCCTTACCA | AGGGTTGTTGTGTATT | 54 / 45 |
| *CD1A* | ATGGAGAAAAGGTGTTAGTTTGTAT | ATATATTCTCTTCCCCTATCTCTTCAACCC | AGAAAAGGTGTTAGTTTG | 60 / 45 |
| *GADD45a* | TTGGGAGGTAGAGGTTTAATTAGTGT | CCCCAACTCCTCCTCCTATACCAA | TGGGAGGTAGAGGTTTAATTA | 55.7 / 40 |
| *CDKN2A* | AGGGGTTGGTTGGTTATTAG | CTACCTACTCTCCCCCTCTC | GGTTGGTTATTAGAGGGT | 58 / 40 |

Table 2S. CpG Locations

| Gene | # CpGs | CpG Locations (hg19) | Distance to TSS (bp) |
| --- | --- | --- | --- |
| *CCNA1* | 4 | Chr13: 37,006,842; 37,006,858; 37,006,872; 37,006,888 | 201 |
| *CD1A** | 2 | Chr1: 158,223,921; Chr1: 158,223,934 | 17 |
| *NDN* | 3 | Chr15: 23,932,338; Chr15: 23,932,371; Chr15: 23,932,374 | 105 |
| *DCC* | 5 | Chr18: 49,868,086; Chr18: 49,868,092; Chr18: 49,868,101; Chr18: 49,868,107; Chr18: 49,868,110 | 1217 |
| *GADD45a* | 5 | Chr1: 65150930; Chr1: 68150934; Chr1: 68150393; Chr1: 68150944; Chr1: 68150949 | 27 |
| *p16* | 4 | Chr9: 21965022; Chr9: 21965027; Chr9: 21965029; Chr9: 21965033 | 128 |

*although these sites are in a CpG poor region, they overlap with those found on the 450K

Table 3S. Quartile and Interquartile Ranges

| Gene | Quartile 1 | Quartile 2 | Quartile 3 | Quartile 4 | IQR* |
| --- | --- | --- | --- | --- | --- |
| *CCNA1* | (3.0, 17.8) | (17.9, 26.0) | (26.1, 37.3) | (37.4,90.5) | (17.8, 37.3) |
| *CD1A* | (21.4, 56.2) | (56.3, 69.8) | (69.9, 81.1) | (81.2, 93.9) | (56.2, 81.1) |
| *NDN* | (10.8, 36.5) | (36.6, 44.2) | (44.3, 54.0) | (54.1, 84.9) | (36.5, 54.0) |
| *DCC* | (3.0, 19.9) | (20.0, 33.0) | (33.1, 50.0) | (50.1, 85.8) | (19.9, 50.0) |
| *GADD45a* | (0, 1.1) | (1.2, 1.4) | (1.5, 1.9) | (2.0, 16.0) | (1.1, 1.9) |
| *p16* | (0, 1.4) | (1.5, 2.3) | (2.4, 3.7) | (3.8, 70.1) | (1.4, 3.7) |

*Interquartile range

**Figure Legends**

**Figure 1S.** *Comparison of survival and recurrence outcomes by HPV (+) oropharyngeal (OP) and HPV (+) non-OP patients. Significant difference between groups is determined by a univariable Cox Proportional Hazards model for each outcome with a p-value <0.05. a) Probabilities of overall survival time did not differ for OP HPV (+) patients and non-OP HPV (+) patients (p-value= 0.71). b) Probabilities of recurrence/persistence free time did not differ for OP HPV (+) patients and non-OP HPV (+) patients (p-value= 0.59)*

**Figure 2S**. *Kaplan-Meier curves of overall survival time and recurrence-free survival time for each methylation marker divided categorized into quartiles.*

Figure 3S. *Kaplan- Meier curves of overall survival time for each methylation marker, stratified by HPV status for overall survival time.*

Figure 4S. *Kaplan- Meier curves of overall survival time for each methylation marker, stratified by HPV status for recurrence-free survival time.*
